# Balloon-Assisted Tracking to Overcome Radial Spasm during Transradial Coronary Angiography: A Case Report

**DOI:** 10.1155/2014/214310

**Published:** 2014-01-08

**Authors:** N. J. W. Verouden, F. Kiemeneij

**Affiliations:** ^1^Department of Cardiology, B2-137, Academic Medical Center-UvA, Meibergdreef 9, 1105 AZ Amsterdam, The Netherlands; ^2^Department of Cardiology, Tergooi, Rijksstraatweg 1, 1261 AN Blaricum, The Netherlands

## Abstract

Spasm of the radial artery is the most important cause of failure to perform coronary angiography via the transradial approach. Spasmolytic cocktail may prevent radial artery spasm but is relatively contraindicated in patients with aortic stenosis or diminished left ventricular function. In this case report we describe a recently published technique to overcome severe radial spasm during transradial coronary angiography in a patient with moderate aortic valve stenosis.

## 1. Introduction

After a two-decade evolution, the transradial approach (TRA) in coronary angiography and percutaneous coronary intervention (PCI) has become a viable and attractive alternative for the femoral approach [1–4]. The increased adoption of the TRA originates from high procedural success, reduced risk for major access site related bleeding complications, lower mortality, increased patient comfort, and cost reduction [5–7].

One of the most encountered problems is radial artery spasm. In a multicenter registry containing over 1900 transradial procedures, the incidence of radial spasm was 2.7%, with multiple puncture attempts and use of larger introducer sheaths (7F) being independent predictors of radial spasm [8]. Another prospective study reported female gender as an independent contributor to the incidence of radial spasm [9]. Furthermore, spasm can be triggered by excessive manipulation of intra-arterial wires and guides, especially if there is some mismatch in diameter between a small radial artery and a large bore catheter.

Radial artery spasm can be prevented by administration of intraarterial spasmolytic cocktails [10]. However, in some instances these cocktails may result in hypotension and bradycardia. Patients with a significant aortic stenosis have a fixed stroke volume and therewith are at high risk of refractory and life threatening hypotension after administration of spasmolytic cocktail.

In this case report, we describe a recently published technique [11] to overcome severe radial spasm in a patient with moderate to severe aortic valve stenosis who underwent TRA coronary angiography.

## 2. Case

A 70-year-old male patient was referred for coronary angiography because of chest pain on exertion. In preceding years, he visited our outpatient department for echocardiographic follow-up of a moderate aortic valve stenosis. During the last visit he expressed typical anginal complaints while echocardiographic evaluation of the aortic valve displayed a stable function.

**Figure 1:**
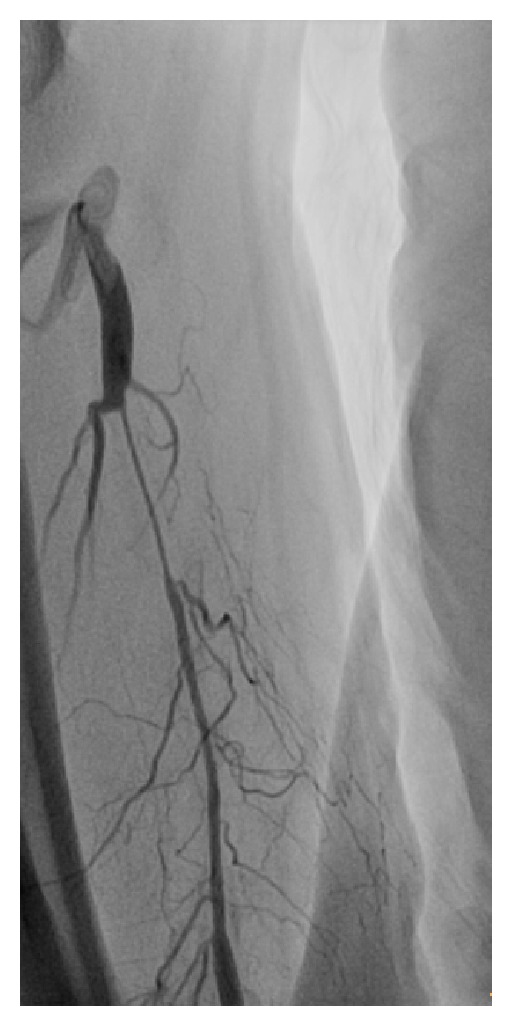


Patient underwent coronary angiography via right radial access. After uneventful puncture and cannulation of a 5F introducer sheath (Arrow, Arrow International, Inc.) 5000 IU of heparin was administered. Initially, no spasmolytic cocktail was used because of patient's moderate aortic stenosis. A 5F JL catheter (Medtronic Inc., Minneapolis, MN. USA) over a 0.035′′ standard guide wire could uneventfully cannulate the left main ostium. After visualization of the left coronary artery, over the wire exchange for a 5F JR catheter resulted in severe patient discomfort of the forearm, resulting from spasm. Half-dose spasmolytic cocktail (5 mg of verapamil plus 200 ug nitroglycerine in 10 mL of normal saline) did not permit the JR catheter to pass and resulted in a significant fall of intra-arterial blood pressure. Visualization of the radial revealed severe and refractory spasm (Figure 1).

**Figure 2:**
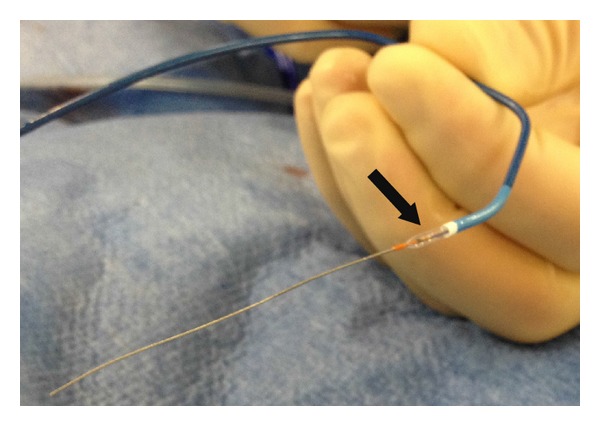
The BAT assembly: Kimny guiding catheter, protruding balloon (black arrow), and Sion guide wire.

In order to cross the spastic segment, a 5F Kimny guide (Boston Scientific) was loaded with a 2.0 × 15 mm Mini Trek PTCA catheter (Abbott Vascular) over a 0.014′′ Sion coronary guide wire (Asahi). The balloon catheter, which was kept partially outside the distal end of the guiding catheter, was inflated at 4 bars to ensure fixation of the guiding catheter onto the coronary wire (Figure 2). As such, the guide catheter tapered perfectly over the soft balloon and the balloon over the guide wire. Balloon-assisted tracking (BAT) of the guiding catheter over the 0.014′′ coronary wire resulted in smooth and uncomplicated engagement of the assembly into the ascending aorta in the absence of complaints of the patient. After exchange for the standard 0.035′′ guide wire, subsequent cannulation of the right coronary artery resulted in adequate visualization of this coronary artery.

**Figure 3:**
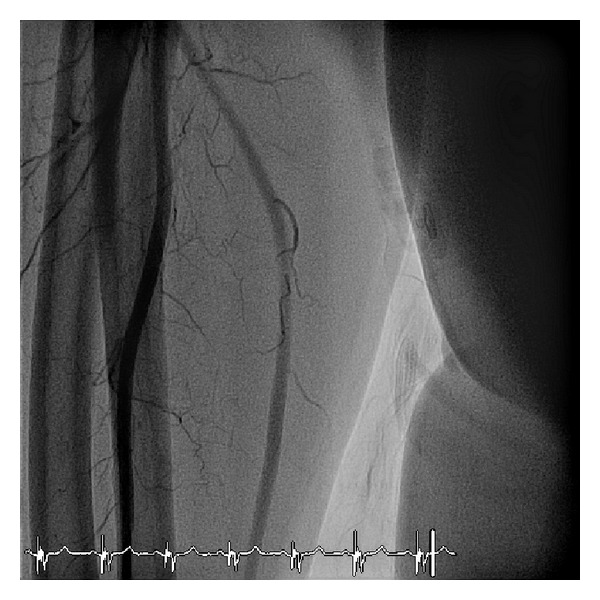


The patient underwent PCI of the ostial lesion of the RCA 1 week later. Radial artery angiography revealed a smooth and undamaged vessel (Figure 3).

## 3. Discussion

The evolution of the TRA in the preceding two decades brought along new procedural difficulties that should be overcome by evolving techniques and equipment. As in above-described case, spasm of the radial artery are a major obstacle to a successful TRA and may result in catheter-induced perforation or conversion to a transfemoral approach.

The tunica media of the muscular radial artery is comprised of concentric layers of smooth muscle cells. Both circulating catecholamines and mechanical stimuli (sheath introduction, wire, and catheter manipulation) may cause smooth muscle cell contraction and hereby spasm of the radial artery. Forced cannulation of a spastic radial artery may cause the catheter tip to brush the endothelium, which may aggravate spasm and cause vascular damage. BAT of the guiding catheter was recently described by Patel and colleagues [11] to overcome complex arm vasculature like small size, tortuosity, and sclerosis. Key factor of success of this technique is the conical shape of the partly protruding balloon with its coated surface, which gives the assembly a tapered tip and a smooth surface. Low pressure balloon inflation (3 bar) keeps the tip more flexible and hence suitable for extreme tortuosity and obstructions of the peripheral arteries. Better pushability to overcome spasm or small vessel diameter can be obtained by medium pressure balloon inflation (6 bar) [12].

In conclusion, BAT was successful in this patient with radial artery spasm refractory to low dose spasmolytics.

## References

[1] Bertrand OF, Rao SV, Pancholy S (2010). Transradial approach for coronary angiography and interventions: results of the first international Transradial practice survey. *Journal of the American College of Cardiology*.

[2] Feldman DN, Swaminathan RV, Kaltenbach LA (2013). Adoption of radial access and comparison of outcomes to femoral access in percutaneous coronary intervention: an updated report from the national cardiovascular data registry (2007–2012). *Circulation*.

[3] Kiemeneij F, Laarman GJ, Odekerken D, Slagboom T, van der Wieken R (1997). A randomized comparison of percutaneous transluminal coronary angioplasty by the radial, brachial and femoral approaches: the access study. *Journal of the American College of Cardiology*.

[4] Hamon M, Pristipino C, Di Mario C (2013). Consensus document on the radial approach in percutaneous cardiovascular interventions: position paper by the European Association of Percutaneous Cardiovascular Interventions and Working Groups on Acute Cardiac Care** and Thrombosis of the European Society of Cardiology. *EuroIntervention*.

[5] Cooper CJ, El-Shiekh RA, Cohen DJ (1999). Effect of transradial access on quality of life and cost of cardiac catheterization: a randomized comparison. *American Heart Journal*.

[6] Jolly SS, Yusuf S, Cairns J (2011). Radial versus femoral access for coronary angiography and intervention in patients with acute coronary syndromes (RIVAL): a randomised, parallel group, multicentre trial. *The Lancet*.

[7] Romagnoli E, Biondi-Zoccai G, Sciahbasi A (2012). Radial versus femoral randomized investigation in ST-segment elevation acute coronary syndrome: the RIFLE-STEACS (Radial Versus Femoral Randomized Investigation in ST-Elevation Acute Coronary Syndrome) study. *American College of Cardiology*.

[8] Goldsmit A, Kiemeneij F, Gilchrist IC (2013). Radial artery spasm associated with transradial cardiovascular procedures: results from the RAS registry. *Catheterization and Cardiovascular Interventions*.

[9] Gorgulu S, Norgaz T, Karaahmet T, Dagdelen S (2013). Incidence and predictors of radial artery spasm at the beginning of a transradial coronary procedure. *Journal of Interventional Cardiology*.

[10] Kiemeneij F, Vajifdar BU, Eccleshall SC, Laarman G, Slagboom T, van der Wieken R (2003). Evaluation of a spasmolytic cocktail to prevent radial artery spasm during coronary procedures. *Catheterization and Cardiovascular Interventions*.

[11] Patel T, Shah S, Pancholy S (2013). Balloon-assisted tracking of a guide catheter through difficult radial anatomy: a technical report. *Catheterization and Cardiovascular Interventions*.

[12] Patel T, Shah S, Pancholy S (2013). Balloon-assisted tracking: a must-know technique to overcome difficult anatomy during transradial approach. *Catheterization and Cardiovascular Interventions*.

